# A pilot study to evaluate the quality of care in oral anticoagulant and antiplatelet use in patients with permanent atrial fibrillation in Irish general practice

**DOI:** 10.1017/S1463423623000622

**Published:** 2024-04-25

**Authors:** Sarah McErlean, John Broughan, Geoff McCombe, Ronan Fawsitt, Walter Cullen, Joe Gallagher

**Affiliations:** 1 Department of General Practice, UCD School of Medicine, University College Dublin, Belfield, Dublin 4, Ireland; 2 Palms General Practice Surgery, The Avenue, Gorey, Co. Wexford, Ireland; 3 Summerhill Family Practice, Summerhill, Dublin 1, Ireland; 4 Mater Misericordiae Hospital, Dublin 7, Ireland

**Keywords:** general practice, atrial fibrillation, quality of care, anticoagulant, antiplatelet

## Abstract

**Aim:**

The aim of this pilot study is to determine the pattern of oral anticoagulant and antiplatelet use in patients with permanent atrial fibrillation (AF) in Irish general practice.

**Background:**

Worldwide, AF is the most common sustained cardiac arrhythmia in adults and poses a significant burden to patients, physicians and healthcare systems. There is a five-fold increased risk of stroke with AF, and AF-related strokes are associated with higher levels of both morbidity and mortality compared to other stroke subtypes. Thankfully, appropriate use of oral anticoagulation (OAC) for AF can reduce the risk of stroke by up to 64%. However, we know that patients are commonly undertreated with OAC, prescribed inappropriate doses of OAC and have prolonged use of an antiplatelet agent in addition to OAC without indication.

**Methods:**

A descriptive, cross-sectional observational study was undertaken. Proportionate sampling was used across 11 practices from the Ireland East practice-based research network. The general practitioners completed a report form on each patient provided by the research team by undertaking a retrospective chart review.

**Findings:**

Eleven practices participated with a total number of 1855 patients with AF. We received data on 153 patients.

The main findings from this pilot project are that:

1. 11% of patients were undertreated with OAC
2. 20 % of patients were on an incorrect non-vitamin K antagonist oral anticoagulant dose
3. 28 patients (18%) were inappropriately prescribed combination antithrombotic therapy

Undertreatment and underdosing of OAC expose patients to higher risk of thromboembolic events, bleeding and all-cause mortality. Prolonged combination antithrombotic therapy is associated with serious increased risk of bleeding with no additional stroke protection. This pilot project highlights several gaps between guidelines and clinical practice. By identifying these areas, we hope to develop a targeted quality improvement intervention using the electronic health records in general practice to improve the care that those with AF receive.

## Introduction

Worldwide, atrial fibrillation (AF) is the most common sustained cardiac arrhythmia in adults and poses a significant burden to patients, physicians and healthcare systems (Hindricks *et al.*, 2021). Approximately 1 in 3 European adults over the age of 55 will develop AF (Hindricks *et al.*, 2021). Data from the Irish Longitudinal Study on Ageing (TILDA) estimate the prevalence of AF in Ireland to be 3.2% in those over the age of 50 years and nearing 20% in men over 80 years of age (Frewen *et al.*, 2013). They have predicted this prevalence to increase three-fold by 2040 (Frewen *et al.*, 2013). This rising prevalence is due to an aging population, more vigorous searching for undiagnosed AF and the increasing burden of comorbidities including hypertension, diabetes, heart failure, coronary artery disease (CAD), chronic kidney disease, obesity and obstructive sleep apnea (OSA) (Hindricks *et al.*, 2021). Modifiable risk factors are potent contributors to AF development and progression. (Hindricks *et al.*, 2021).

Despite its prevalence, management of AF remains suboptimal both in Ireland and across the world (Hannon *et al.*, 2009; Frewen *et al.*, 2013).

The European Society of Cardiology (ESC) highlights that anticoagulation is a key priority when providing AF care in their Atrial Fibrillation Better Care pathway (Hindricks *et al.*, 2021). There is a five-fold increased risk of stroke with AF, and AF-related strokes are associated with higher levels of both morbidity and mortality compared to other stroke subtypes (Jones *et al*., 2018; Hindricks *et al.*, 2021). In Ireland, stroke is the leading cause of acquired disability (Irish Heart Foundation, 2010) and the second leading cause of death (Institute for Health Metrics and Evaluation, 2019).

Thankfully, appropriate use of anticoagulation for AF can reduce the risk of stroke by up to 64%; one of the highest impacts of any medical intervention for a common long-term condition (Hart *et al*., 2007). However, we know that patients do not receive adequate monitoring of anticoagulants (Kassianos *et al.*, 2013; Murphy *et al.*, 2020; Wijtvliet *et al.*, 2020) and are undertreated with anticoagulation (Hannon *et al.*, 2009; Frewen *et al.*, 2013; Shantsila *et al.*, 2015; Lacoin *et al.*, 2017; Robson *et al.*, 2018; Murphy *et al.*, 2020). One of the main barriers to appropriate prescribing is the underestimation of stroke risk in young patients and an overestimation of bleeding risk in elderly patients (Lacoin *et al.*, 2017). Recent figures from the United Kingdom show that only 67% of patients with AF are treated with oral anticoagulation (Lacoin *et al.*, 2017). Those treated with Warfarin are only in the therapeutic range 40% of the time (López-López *et al.*, 2017), and patients are frequently prescribed a suboptimal non-vitamin K antagonist oral anticoagulant (NOAC) dose (Steinberg *et al.*, 2018). In the United States of America (USA) nearly 60% of reduced-dose NOAC regimens do not follow Food and Drug Administration (FDA) recommendations (Steinberg *et al.*, 2018). Inappropriate dosing may be associated with significant adverse events such as bleeding, thromboembolic events and all-cause mortality (Steinberg *et al.*, 2018).

Combination antithrombotic therapy, which refers to an anticoagulant in addition to an antiplatelet agent, after an event such as a myocardial infarct or coronary artery bypass graft, is rarely indicated for more than 1 year in duration, and for many, the recommended duration of combination antithrombotic therapy is 6 months (Xie *et al.*, 2022). If a patient is taking an antiplatelet agent for secondary prevention and develops AF; if they are > 1 year since event and have stable disease then they should be suitable for oral anticoagulant (OAC) monotherapy.

It has been shown that there is no difference in cardiovascular mortality or stroke with prolonged use of combination antithrombotic therapy compared to OAC monotherapy (Shakir *et al.*, 2022). However, combination antithrombotic therapy (which includes an anticoagulant and an antiplatelet agent) significantly increases the risk of fatal and non-fatal bleeding; upper gastrointestinal bleeding (UGIB) in particular (van Rein *et al.*, 2019; Yasuda *et al.*, 2019; Shakir *et al.*, 2022).

In Ireland, general practitioners (GPs) can initiate OAC in patients with stable, new-onset atrial fibrillation based on their CHA2DS2-VASc score and are encouraged to start NOAC therapy rather than Warfarin (Irish College of General Practitioners Quick Reference Guide (ICGP QRG), 2020). The use of NOACs in Ireland is increasing (Kennedy *et al.*, 2022). Once a patient is on OAC (even if this is initiated in secondary care), the GP will be responsible for the ongoing monitoring and prescription of these drugs. Certain patients will attend both primary and secondary care and require an integrated approach to management.

A ‘General Medical Service’ (GMS) card or a ‘Doctor Visit Card’ (DVC) entitles patients in Ireland to free GP care. These are awarded to all patients over 70 years, under 6 years of age and those below a certain income level based on means testing. These patients are eligible for a biannual structured chronic disease management (CDM) review for AF. This provides an opportunity to manage comorbidities, control risk factors and ensure that the treatment strategy for the patient is optimal; paying particular attention to the presence of anticoagulation and the dose of anticoagulant based on renal function. This was introduced in March 2020.

The success of the CDM program thus far can be seen in the improvements made in the risk factor profile of AF patients (HSE, 2023). This program has resulted in increased physical activity levels, reduced alcohol intake, reduced blood pressure and reduced BMI (HSE, 2023). We know that alcohol increases the bleeding risk in anticoagulated patients and increases the incidence of AF. The recent Alcohol-AF trial demonstrated that even moderate drinkers have a reduction in AF recurrence, symptoms and time spent in AF with abstinence (Voskoboinik *et al.*, 2020).

Tackling obesity in addition to other CVD risk factors can reduce AF incidence, progression, recurrence and symptoms. Weight loss of ≥10% has been shown to reverse the progressive nature of AF, with those who achieve this being more likely to revert to sinus rhythm or paroxysmal AF (Middeldorp *et al.*, 2018).

The aim of this pilot study is to determine the pattern of OAC and antiplatelet use in patients with permanent AF in Irish general practice.

## Methods

### Study design

A descriptive, cross-sectional observational study was undertaken.

### Setting

The 14 practices in the Ireland East practice-based research network were invited to participate in this pilot study via a letter of invitation. The GPs were invited to a virtual information evening run by the investigation team in January 2022 to distribute written and oral information on the study and provide a platform to answer questions. The research team then contacted the GPs via email to provide participant information leaflets and consent forms in April 2022.

The GPs were asked to return a signed consent form and the total number of patients with permanent AF in their practice if they were happy to participate. We used proportionate sampling to generate a sample size for each practice.

The research team then provided report forms to complete and a notice to be displayed in their practices informing their patients that this study was taking place.

Each participating practice used a random number generator to generate a list of patients from the practice to include in the study.

Data were collected retrospectively on these patients in May and June 2022 and recorded on a report form provided by the research team. This was undertaken by the GP and data controller in each participating practice to comply with general data protection regulations.

The GPs recorded anonymous data only. Data collected included demographics, risk factors for AF, medications, investigations and AF management details. The soft copy report forms were returned to the research team at University College Dublin (UCD). This data was then cleaned, pooled and inputted into a secure database.

### Participants

All practices in the Ireland East practice-based research network were invited to participate.

They identified patients in the practice with permanent AF.

Inclusion criteria:

- Age ≥ 18 years
- Permanent AF detected on electrocardiogram, Holter recording or event recorder
- Active in practice software and has attended the practice in the last 24 months

Exclusion criteria:

- No electrocardiographic objectified AF

### Data sources

The GPs undertook a retrospective chart review of the electronic patient record in the GP management software system. Anonymous details were recorded manually on a report form provided by the research team.

### Bias

Each practice used a random number generator to create a list of patients to include to reduce sampling bias.

### Study size

As this was a pilot study, a formal sample size was not calculated (Billingham *et al*., 2013). Based on previous work, it was estimated that 12 per group would be sufficient (Julious, 2005; van Belle, 2008). We estimated that 80% of the practices would complete the study (11 practices). Therefore we sought to recruit a minimum of 132 patients using proportional sampling.

### Statistical methods

The secure database RedCap was used for data storage and analysis. The Statistical Package for Social Sciences program was used for statistical analysis. The Chi square test of independence was used to analyse categorical variables. Missing data were either excluded from analysis or calculated based on information provided, for example, creatinine clearance.

### Ethical approval

Granted by Irish College of General Practitioners Research Ethics Committee.

## Results

### Participants

Eleven practices consented to participate in this pilot study. These 11 practices had a total number of 1855 patients with AF. We received data on 153 patients proportionally sampled across these 11 practices.

### Descriptive data

The demographic details can be seen in Table 1. Most patients – 68% were male (*N* = 104) and the mean age was 76 years.

**Table 1.** Patient demographics

Demographics							
Gender	Male	Female	Missing				
	104	46	3				
Age	Min	Max	Mean	Median	Missing		
	48 yrs	96 yrs	76 yrs	78 yrs	3		
Medical Card Status	GMS	DVC	Private	Missing			
	116	11	17	9			
CDM Review	Yes	No	Missing				
	115	37	1				
Smoking Status	Current	Ex-Smoker	Never Smoker	Missing			
	17	52	75	9			
Alcohol Intake (units/week)	Min	Max	Mean	Missing			
	0	140	4.8	24			
BMI kg/m^2^	Min	Max	Mean	Median	Missing		
	17	45	29.2	28.4	30		
Co-Morbidities	Hypertension	CAD	Diabetes	Stroke/TIA	PVD	OSA	Hyperthyroid
	79	57	41	23	7	6	3

GMS = General Medical Services; DVC = Doctor Visit Card; CDM = Chronic Disease Management; CAD = Coronary Artery Disease; TIA = Transient Ischaemic Attack; PVD = Peripheral Vascular Disease; OSA = Obstructive Sleep Apnoea.

The majority of patients had a GMS card or a DVC (83% *N* = 127). 115 (75%) patients had CDM review in their practice.

Hypertension (52%), cardiovascular disease (37%), diabetes (27%) and cerebrovascular disease (15%) were the most common comorbidities in this group.

The mean BMI was 29.2 kg/m^2^ (Range 17–45 kg/m^2^) with 36.4% categorized as obese with a BMI > 30 kg/m^2^. The mean weekly alcohol intake was 4.8 units.

### Main results

Of the records analysed, 14% of AF patients (21 patients) were not on any anticoagulation. Based on the patient’s CHA2DS2-VASc score of 1 in a male or 2 in a female, OAC was indicated in the majority of these patients (17 patients). Of the patients without OAC at high risk of stroke (CHA2DS2-VASc ≥ 2), 11 out of 17 were on an antiplatelet agent. Nine of these were on aspirin for secondary prevention and two did not have a clear indication.

**Figure 1.**
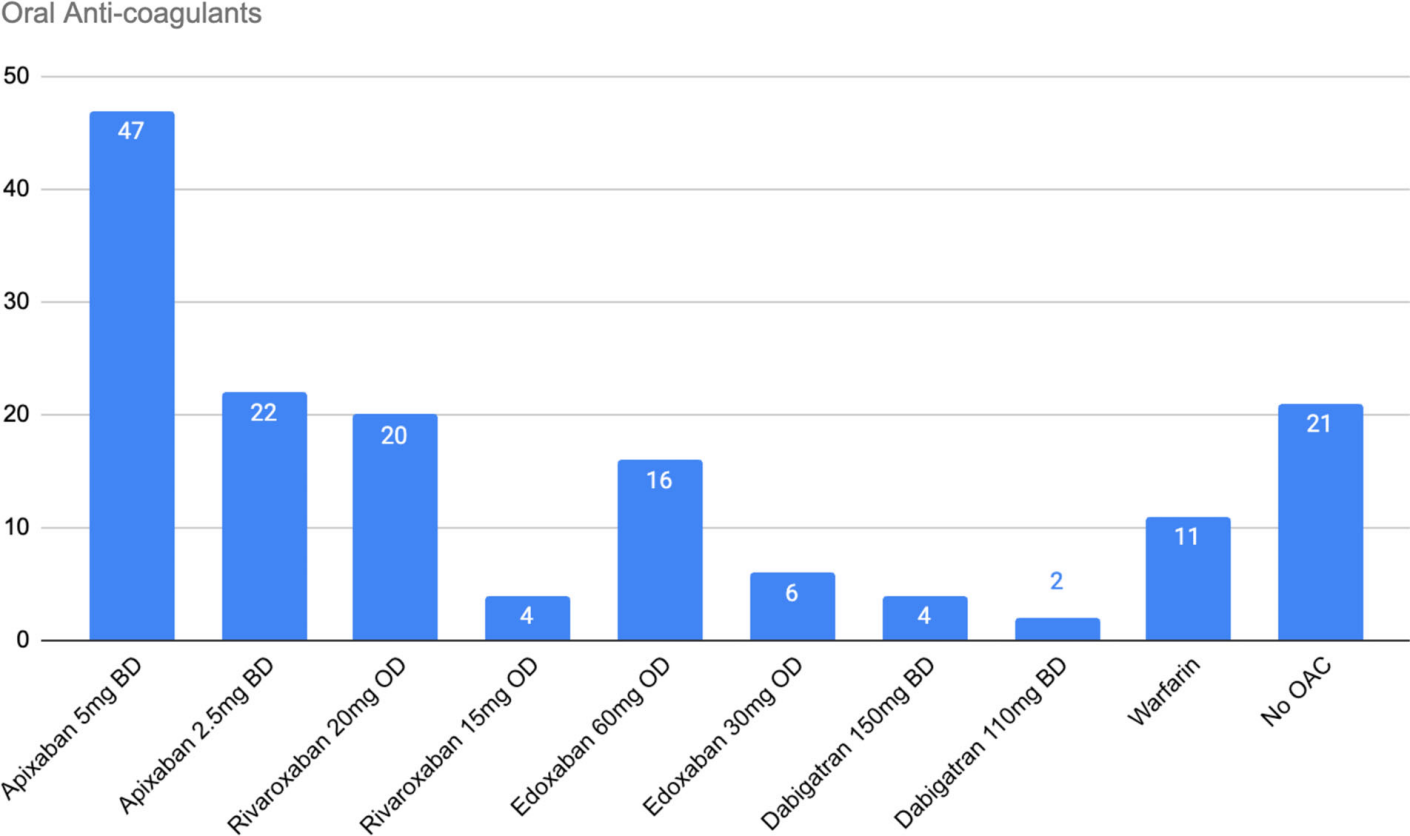
Oral anticoagulants.

There were 132 patients on OAC. The most common OAC was Apixaban (52.3%), followed by Rivaroxaban (18.2%), Edoxaban (16.7%), Warfarin (8.3%) and Dabigatran (4.5%). See Figure 1.

There were 121 patients on NOACs. We analysed these patient records to determine if they were on the correct dose of the NOAC. Sixteen records were incomplete and did not have key information recorded, such as age/weight/renal function, so we could not determine if the dosage was correct or not.

**Figure 2.**
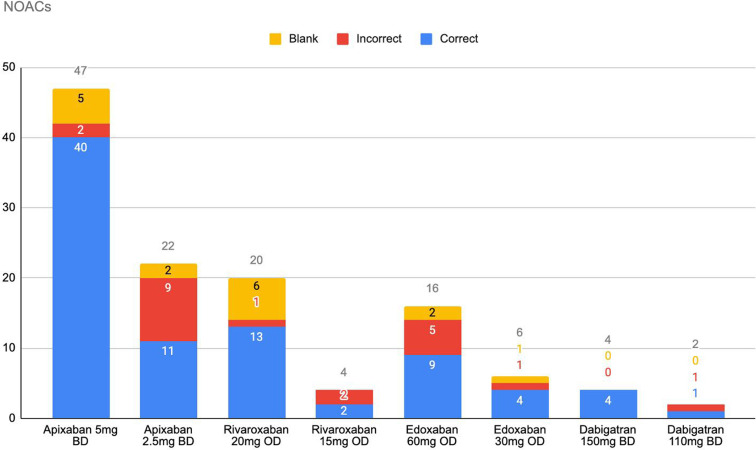
Non-vitamin K antagonist oral anticoagulant doses.

Of the 105 records analysed, 84 patients (80%) were on the correct dose and 21 patients (20%) were on an incorrect dose. Thirteen patients should have been on a higher dose of OAC and eight met the criteria for dose reduction. The most common error was the inappropriate prescription of the lower dose of Apixaban; 2.5 mg BD. See Figure 2.

Of those who were on the correct dose of NOAC, 61 were male and 23 were female. Of those on an incorrect dose, 10 were male and 11 were female. A chi-square test of independence was performed to evaluate the relationship between correct NOAC dose and gender. The relationship between these variables was significant, *X* ^2^ (1, *N =* 105) = 4.795, *P* < 0.05 (*P* = 0.029). Males were more likely to be on the correct NOAC compared to females.

In this pilot study, 41 patients were taking an antiplatelet agent. In total, 32 of these patients were taking an antiplatelet agent for secondary prevention and 9 patients did not have a clear indication. There were no patients taking dual antiplatelet therapy. The majority of these patients were taking aspirin 75 mg (90.2%). Of these, 29 were taking both an antiplatelet agent and OAC. Only 1 of these patients had percutaneous coronary intervention (PCI)/coronary artery bypass graft (CABG) within the last year meaning that 28 patients were on combination antithrombotic therapy without an indication. Combination antithrombotic therapy was defined as concomitant prescription of an antiplatelet agent (aspirin, clopidogrel, prasugrel or ticagrelor) and an anticoagulant (warfarin, apixaban, rivaroxaban, edoxaban or dabigatran). Coronary artery disease was present in 64% (*N* = 18) of patients who were inappropriately prescribed combination antithrombotic therapy.

A chi-square test of independence was performed to evaluate the relationship between potentially inappropriate combination antithrombotic therapy and a history of coronary artery disease. The relationship between these variables was significant, *X* ^2^ (1, *N =* 153) = 10.713, *P* = < 0.001. Those with CAD were more likely to be on inappropriate combination of antithrombotic therapy.

## Discussion

### Key results

The key results from this project are that:

1. 11% of patients were undertreated with OAC
2. 20% of patients were on an incorrect NOAC dose
3. 28 patients (18%) were inappropriately prescribed combination antithrombotic therapy

### Limitations

There are several limitations to this retrospective observational study.

The report form that we devised did not ask explicitly if there was an absolute contraindication to OAC, a history of major bleeding or another indication for cessation of therapy. As the information was inputted manually, some of the data were not complete.

We did not ask if there specific reason for an antiplatelet agent in addition to OAC, for example, high-risk disease/recommendation from interventional cardiology/hematological indication.

We also did not assess the international normalised ratio value for those on Warfarin, therefore, we were unable to determine if the dose was therapeutic.

### Interpretation

#### Patients undertreated with OAC

This study found that 11% of patients at high risk of stroke were not anticoagulated. Of the patients without OAC at high risk of stroke (CHA2DS2-VASc ≥ 2), 11 out of 17 were on an antiplatelet agent. Nine of these were on aspirin for secondary prevention and two did not have a clear indication. This suggests that these patients may have been started or maintained on antiplatelet monotherapy as stroke prevention in the setting of AF. However, there is no role for antiplatelet agents as monotherapy in AF for stroke prevention (Hindricks *et al.*, 2021; NICE, 2021) and aspirin is associated with a significant risk of bleeding.

Undertreatment with anticoagulation is a common problem in AF care across the world and the rates of this pilot study are comparable to other work in this area. A cross-sectional study from the UK in 2017 demonstrated that only 67% of patients with AF were receiving anticoagulation (Flaker *et al.*, 2012; Lacoin *et al.*, 2017). The GARFIELD – AF registry reports that 71.1% of patients with AF are on OAC and a recent randomised trial from the Netherlands reported an anticoagulation rate of 81% (Wijtvliet *et al.*, 2020; Bassand *et al.*, 2021).

We also know that patients with known AF who present with acute ischemic stroke (the most serious consequence of AF) have a high prevalence of inadequate anticoagulation preceding their stroke (Xian *et al.*, 2017). This was shown in an Irish context where a Dublin-based prospective study on stroke in 2009 showed that only 32% of patients with known AF and prior stroke were anticoagulated at the time of their recurrent ischemic stroke (Hannon *et al.*, 2009). Echoing these findings, a systematic review in 2010 of 54 studies from 11 countries found a consistent pattern of OAC underuse in AF patients with an elevated stroke risk (Ogilvie *et al.*, 2010).

One of the main barriers to appropriate prescribing is the overestimation of bleeding and falls risk in elderly patients (Lacoin *et al.*, 2017; Xian *et al.*, 2017). The absolute contraindications to OAC are quite limited. They include serious active bleeding, severe thrombocytopenia <50 platelets/uL or recent high-risk bleeding, for example, intra-cranial hemorrhage (Hindricks *et al.*, 2021). Age and falls risk are certainly not contraindications in their own right.

#### Patients on incorrect NOAC dose

Of those who were on a NOAC, 20% (21 patients) were on an incorrect dose. Interestingly, 13 patients should have been on a higher dose and eight met the criteria for dose reduction.

It is a common research finding that patients are on an incorrect NOAC dose; in particular inappropriate dose reduction. A retrospective trial in Boston demonstrated that 14.7% of patients on a reduced-dose NOAC had no indication for such a reduction (Barra *et al.*, 2016). An Australian study of NOAC prescribing showed that 34% of patients were on an incorrect NOAC dose; of these 22% were overdosed and 38% were underdosed (Pattullo *et al.*, 2016). In the USA, nearly 60% of reduced-dose NOAC regimens do not follow FDA recommendations (Steinberg *et al.*, 2018).

Inappropriate dosing can be associated with significant adverse events such as bleeding, thromboembolic events and all-cause mortality (Barra *et al.*, 2016; Steinberg *et al.*, 2018). Undertreating these patients exposes them to the increased risk of bleeding and yet does not offer them protection against embolic strokes (Barra *et al.*, 2016; Steinberg *et al.*, 2018). In a large retrospective study from the USA, we saw that inappropriate under dosing was associated with a nearly 5-fold increased risk of stroke in apixaban-treated patients with bleeding rates comparable to the standard dose (Yao *et al.*, 2017).

It is a paradox that women are deemed at higher risk of embolic stroke and will score an extra point on the CHA2DS2-VASc score and yet are less likely to be on an appropriate dose of OAC as illustrated in this pilot study. This finding is not unique and has been highlighted in several other research studies (Steinberg *et al.*, 2016; Lee *et al.*, 2018). It has also been shown that women are less likely to be prescribed OAC in AF and less likely to be maintained on this treatment (Thompson *et al.*, 2017; Ferroni *et al.*, 2019; Tzeis *et al.*, 2021).

The reasons for this discrepancy are not entirely clear but we do know that a history of minor bleeding is associated with NOAC underdosing (Caso *et al.*, 2023) and women tend to present more frequently with adverse drug reactions such as this (Giner-Soriano *et al.*, 2023). It may also be due to renal function assessment; using eGFR rather than creatinine clearance can overestimate renal function in females (Howard *et al.*, 2017).

The four NOACs available to us have different dosages and different criteria for dose reduction making identification of the correct dose complicated and challenging (Steffel *et al.*, 2021). All NOACs have some degree of renal clearance. Apixaban includes age, weight and serum creatinine in its dose reduction criteria, whereas reduction of the other three NOACs (Rivaroxaban, Edoxaban and Dabigatran) is based on creatinine clearance. This variation in dosing guidelines likely contributes to prescribing error (Steinberg *et al.*, 2018). A guide to correct dosing is included in the appendix. Creatinine clearance needs to be calculated using the patient’s age and weight using the Cockcroft-Gault formula. Most laboratories report renal function as an estimation of the glomerular filtration rate (eGFR ml/min) using the Modification of Diet for Renal Disease formula, which can overestimate renal function in older patients with low body weight (Johnson *et al.*, 2012). Estimated GFR produces higher values than Cockcroft & Gault's estimated creatinine clearance at older ages (Schwartz, 2016) and may fail to identify those who should receive reduced-dose NOACs (Dowling *et al.*, 2013). Having renal function reported using a different formula to what is in the prescribing guidelines creates a barrier to appropriate dosing.

#### Inappropriate co prescribing of an antiplatelet with an OAC

Most patients who are on an antiplatelet agent for secondary prevention and then develop AF should be suitable to stop their antiplatelet and continue on OAC alone. The current ESC and National Institute for Health and Care Excellence (NICE) guidelines support the health care provider to stop their antiplatelet agent if they have stable coronary artery disease (>1-year post-event, CABG or PCI) and no symptoms of CAD such as angina. These patients will then remain on OAC monotherapy (Yasuda *et al.*, 2019; Hindricks *et al.*, 2021; NICE, 2021).

In some cases where a patient is unstable or has high-risk disease, they may remain on combination antithrombotic therapy. This should be a specialist-led decision given that combination antithrombotic therapy increases the risk of fatal and non-fatal bleeding (van Rein *et al.*, 2019; Yasuda *et al.*, 2019).

The combination of antiplatelet therapy with OAC in patients who have stable coronary artery disease highlights a gap between guidelines and clinical practice.

In our pilot study, 18% of patients (28 people) were inappropriately prescribed an antiplatelet in addition to their OAC putting these patients at higher risk of bleeding.

Using multiple antithrombotic therapies results in an increased risk of serious bleeding complications; in particular, UGIB (Xie *et al.*, 2022). UGIB is the most common cause of hospital admission for an adverse drug-related event (Pirmohamed *et al.*, 2004). Antiplatelet, anticoagulant use and especially the use of both in combination with older age accounts for a large proportion of these admissions (Pirmohamed *et al.*, 2004). A recent observational study in primary care in the UK has shown that most patients (65.9%) were prescribed combination antithrombotic therapy for an inappropriate prolonged duration of > 12 months (Xie *et al.*, 2022).

A US-based quality improvement study showed that reducing inappropriate co-prescription of aspirin with warfarin was associated with a reduction in bleeding events, major bleeding events and attendance at an emergency department with bleeding (Schaefer *et al.*, 2022). Those on combination antithrombotic therapy were identified and their primary care physician was asked to consider if this co-prescription was necessary and if not then to modify the prescription (Schaefer *et al.*, 2022). Aspirin de-prescribing increased by 50% (Schaefer *et al.*, 2022).

It has been repeatedly shown that there is no difference in cardiovascular mortality or stroke with prolonged use of combination antithrombotic therapy compared to OAC monotherapy (Schaefer *et al.*, 2022; Shakir *et al.*, 2022). In summary, prolonged combination antithrombotic therapy results in more harm than good.

### Generalisability

The results of this pilot study reflect other similar research work from around the world and are likely representative of the type of care provided in general practice as a whole.

### Conclusion

This pilot project has highlighted several gaps between guidelines and clinical practice. By identifying these areas, we hope to develop a targeted quality improvement intervention to improve the care that those with AF receive.

## Appendix A. Dosing table for non-vitamin K antagonist oral anticoagulants (NOACs)

**Table:**

Name	Apixaban	Rivaroxaban	Edoxaban	Dabigatran
Usual dose	5 mg BD	20 mg	60 mg	150 mg BD
Reduced dose	2.5 mg BD	15 mg	30 mg	110 mg BD
Indication for dose reduction	If **2 of 3** factors are present: Age ≥ 80 years Weight ≤ 60 kg Creatinine > 133 µmol/L Or CrCL < 30 ml/min	CrCL < 50 ml/min	Weight ≤ 60 kg CrCL < 50 ml/min Concomitant therapy with strong P-gp inhibitor, for example, dronedarone, ciclosporin, erythromycin, ketoconazole	>80 years High bleeding risk Taking verapamil/amiodarone CrCL < 50 ml/min Creatinine Clearance < 30 ml/min: **Do not use**

All NOACS not recommended CrCL < 15 ml/min.

## References

[ref1] Barra ME , Fanikos J , Connors JM , Sylvester KW , Piazza G and Goldhaber SZ (2016) Evaluation of dose-reduced direct oral anticoagulant therapy. The American Journal of Medicine 129, 1198–1204. 10.1016/j.amjmed.2016.05.041. 27341955

[ref2] Bassand J-P , Apenteng PN , Atar D , Camm AJ , Cools F , Corbalan R , Fitzmaurice DA , Fox KA , Goto S , Haas S , Hacke W , Jerjes-Sanchez C , Koretsune Y , Heuzey JL , Sawhney JP , Oh S , Stępińska J , Cate VT , Verheugt FW , Kayani G , Pieper KS , Kakkar AK and Garfield-Af Investigators (2021) GARFIELD-AF: a worldwide prospective registry of patients with atrial fibrillation at risk of stroke. Future Cardiology, 17, 19–38. 10.2217/fca-2020-0014. 32696663

[ref4] Billingham SA , Whitehead AL and Julious SA (2013) An audit of sample sizes for pilot and feasibility trials being undertaken in the United Kingdom registered in the United Kingdom Clinical Research Network database. BMC Medical Research Methodology 13, 104. 10.1186/1471-2288-13-104. 23961782 PMC3765378

[ref5] Caso V , de Groot JR , Sanmartin Fernandez M , Segura T , Blomström-Lundqvist C , Hargroves D , Antoniou S , Williams H , Worsley A , Harris J , Caleyachetty A , Vardar B , Field P and Ruff CT (2023) Outcomes and drivers of inappropriate dosing of non-vitamin K antagonist oral anticoagulants (NOACs) in patients with atrial fibrillation: a systematic review and meta-analysis. Heart 109, 178–185. 10.1136/heartjnl-2022-321114. 36316100 PMC9872251

[ref6] Dowling TC , Wang ES , Ferrucci L and Sorkin JD (2013) Glomerular filtration rate equations overestimate creatinine clearance in older individuals enrolled in the baltimore longitudinal study on aging: impact on renal drug dosing. Pharmacotherapy: The Journal of Human Pharmacology and Drug Therapy 33, 912–921. 10.1002/phar.1282. PMC3732548 23625813

[ref7] Ferroni E , Gennaro N , Costa G , Fedeli U , Denas G , Pengo V and Corti MC (2019) Real-world persistence with direct oral anticoagulants (DOACs) in naïve patients with non-valvular atrial fibrillation. International Journal of Cardiology 288, 72–75. 10.1016/j.ijcard.2019.04.061. 31043323

[ref8] Flaker GC , Eikelboom JW , Shestakovska O , Connolly SJ , Kaatz S , Budaj A , Husted S , Yusuf S , Lip GY and Hart RG (2012) Bleeding during treatment with aspirin versus Apixaban in patients with atrial fibrillation unsuitable for warfarin: the Apixaban versus acetylsalicylic acid to prevent stroke in atrial fibrillation patients who have failed or are unsuitable for vitamin k antagonist treatment (AVERROES) Trial. Stroke 43, 3291–3297. 10.1161/STROKEAHA.112.664144. 23033347

[ref9] Frewen J , Finucane C , Cronin H , Rice C , Kearney PM , Harbison J and Kenny RA (2013) Factors that influence awareness and treatment of atrial fibrillation in older adults. QJM: Monthly Journal of the Association of Physicians 106, 415–424. 10.1093/qjmed/hct060. 23504411

[ref10] Giner-Soriano M , Prat-Vallverdú O , Ouchi D , Vilaplana-Carnerero C and Morros R (2023) Sex and gender differences in the use of oral anticoagulants for non-valvular atrial fibrillation: a population-based cohort study in primary health care in Catalonia. Frontiers in Pharmacology 14, 1110036. 10.3389/fphar.2023.1110036. 36825151 PMC9941166

[ref11] Hannon N , Sheehan O , Kelly L , Marnane M , Merwick A , Moore A , Kyne L , Duggan J , Moroney J , McCormack PM , Daly L , Fitz-Simon N , Harris D , Horgan G , Williams EB , Furie KL and Kelly PJ (2009) Stroke associated with atrial fibrillation – incidence and early outcomes in the North Dublin population stroke study. Cerebrovascular Diseases (Basel, Switzerland) 29, 43–49. 10.1159/000255973. 19893311 PMC2914401

[ref12] Hart RG , Pearce LA and Aguilar MI (2007) Meta-analysis: antithrombotic therapy to prevent stroke in patients who have nonvalvular atrial fibrillation. Annals of Internal Medicine 146, 857–867. 10.7326/0003-4819-146-12-200706190-00007. 17577005

[ref13] Hindricks G , Potpara T , Dagres N , Arbelo E , Bax JJ , Blomström-Lundqvist C , Boriani G , Castella M , Dan GA , Dilaveris PE , Fauchier L , Filippatos G , Kalman JM , La Meir M , Lane DA , Lebeau JP , Lettino M , Lip GYH , Pinto FJ , Thomas GN , Valgimigli M , Van Gelder IC , Van Putte BP , Watkins CL and ESC Scientific Document Group (2021) 2020 ESC guidelines for the diagnosis and management of atrial fibrillation developed in collaboration with the European Association for Cardio-Thoracic Surgery (EACTS). European Heart Journal 42, 373–498. 10.1093/eurheartj/ehaa612. 32860505

[ref14] Howard M , Lipshutz A , Roess B , Hawes E , Deyo Z , Burkhart JI , Moll S and Shilliday BB (2017) Identification of risk factors for inappropriate and suboptimal initiation of direct oral anticoagulants. Journal of Thrombosis and Thrombolysis 43, 149–156. 10.1007/s11239-016-1435-3. 27757787

[ref15] HSE (2023) *The second report of the structured chronic disease management treatment programme in general practice*, 36. Retrieved from https://www.hse.ie/eng/services/publications/the-second-report-of-the-structured-chronic-disease-management-treatment-programme-in-general-practice.pdf.

[ref16] Institute for Health Metrics and Evaluation (2019) *What causes the most deaths?* Retrieved 7 June 2022 from https://www.healthdata.org/ireland.

[ref17] Irish College of General Practitioners Quick Reference Guide (ICGP QRG) (2020) *Practical use of Direct Oral Anticoagulants (DOACs) in atrial fibrillation in general practice*. ICGP. Retrieved from https://www.icgp.ie/.

[ref18] Irish Heart Foundation (2010) National clinical guidelines and recommendations for the care of people with stroke and transient Ischaemic attack.

[ref19] Johnson DW , Jones GR , Mathew TH , Ludlow MJ , Doogue MP , Jose MD , Langham RG , Lawton PD , McTaggart SJ , Peake MJ , Polkinghorne K , Usherwood T and Australasian Creatinine Consensus Working Group (2012) Chronic kidney disease and automatic reporting of estimated glomerular filtration rate: new developments and revised recommendations. Medical Journal of Australia 197, 222–223. 10.5694/mja11.11329. 22900871

[ref20] Jones N , Hobbs RF and Taylor CJ (2018) Atrial fibrillation and stroke prevention: where we are and where we should be. British Journal of General Practice 68, 260–261. 10.3399/bjgp18X696257 PMC6001982 29853572

[ref21] Julious SA (2005) Sample size of 12 per group rule of thumb for a pilot study. Pharmaceutical Statistics 4, 287–291. 10.1002/pst.185.

[ref22] Kassianos G , Arden C , Hogan S , Dew R and Fuat A (2013) Current management of atrial fibrillation: an observational study in NHS primary care. BMJ Open 3, e003004. 10.1136/bmjopen-2013-003004. PMC3840336 24271019

[ref23] Kennedy C , Gabr A , McCormack J , Collins R , Barry M and Harbison J (2022) The association between increasing oral anticoagulant prescribing and atrial fibrillation related stroke in Ireland. British Journal of Clinical Pharmacology 88, 178–186. 10.1111/bcp.14938. 34131941

[ref24] Lacoin L , Lumley M , Ridha E , Pereira M , McDonald L , Ramagopalan S , Lefèvre C , Evans D and Halcox JP (2017) Evolving landscape of stroke prevention in atrial fibrillation within the UK between 2012 and 2016: a cross-sectional analysis study using CPRD. BMJ Open 7, e015363. 10.1136/bmjopen-2016-015363. PMC5623501 28951401

[ref25] Lee JM , Kim TH , Cha MJ , Park J , Park JK , Kang KW , Shim J , Uhm JS , Kim J , Park HW , Lee YS , Choi EK , Kim CS , Joung B and Kim JB (2018) Gender-related differences in management of nonvalvular atrial fibrillation in an Asian population. Korean Circulation Journal 48, 519. 10.4070/kcj.2017.0389. 29856147 PMC5986752

[ref26] López-López JA , Sterne JAC , Thom HHZ , Higgins JPT , Hingorani AD , Okoli GN , Davies PA , Bodalia PN , Bryden PA , Welton NJ , Hollingworth W , Caldwell DM , Savović J , Dias S , Salisbury C , Eaton D , Stephens-Boal A and Sofat R (2017) Oral anticoagulants for prevention of stroke in atrial fibrillation: systematic review, network meta-analysis, and cost effectiveness analysis. BMJ. 10.1136/bmj.j5058. PMC5704695 29183961

[ref27] Middeldorp ME , Pathak RK , Meredith M , Mehta AB , Elliott AD , Mahajan R , Twomey D , Gallagher C , Hendriks JML , Linz D , McEvoy RD , Abhayaratna WP , Kalman JM , Lau DH and Sanders P (2018) PREVEntion and regReSsive effect of weight-loss and risk factor modification on atrial fibrillation: the REVERSE-AF study. EP Europace 20, 1929–1935. 10.1093/europace/euy117. 29912366

[ref28] Murphy A , Brosnan S , McCarthy S , O'Raghallaigh P , Bradley C and Kirby A (2020) World Café approach: exploring the future vision of oral anticoagulants for patients with atrial fibrillation (AF) in Ireland. BMJ Open 10, e036493. 10.1136/bmjopen-2019-036493. PMC7517561 32973054

[ref29] NICE (2021) Atrial fibrillation: diagnosis and management. Atrial Fibrillation, 47.

[ref30] Ogilvie IM , Newton N , Welner SA , Cowell W and Lip GY (2010) Underuse of oral anticoagulants in atrial fibrillation: a systematic review. The American Journal of Medicine 123, 638–645.e4. 10.1016/j.amjmed.2009.11.025. 20609686

[ref31] Pattullo CS , Barras M , Tai B , McKean M and Donovan P (2016) New oral anticoagulants: appropriateness of prescribing in real-world setting: prescribing new oral anticoagulants. Internal Medicine Journal 46, 812–818. 10.1111/imj.13118. 27087277

[ref32] Pirmohamed M , James S , Meakin S , Green C , Scott AK , Walley TJ , Farrar K , Park BK and Breckenridge AM (2004) Adverse drug reactions as cause of admission to hospital: prospective analysis of 18 820 patients. BMJ 329, 15–19. 10.1136/bmj.329.7456.15. 15231615 PMC443443

[ref34] Robson J , Homer K , Ahmed Z and Antoniou S (2018) Variation in anticoagulation for atrial fibrillation between English clinical commissioning groups: an observational study. British Journal of General Practice 68, e551–e558. 10.3399/bjgp18X697913. PMC6058620 29970397

[ref35] Schaefer JK , Errickson J , Gu X , Alexandris-Souphis T , Ali MA , Haymart B , Kaatz S , Kline-Rogers E , Kozlowski JH , Krol GD , Shah V , Sood SL , Froehlich JB and Barnes GD (2022) Assessment of an intervention to reduce aspirin prescribing for patients receiving warfarin for anticoagulation. JAMA Network Open 5, 13. 10.1001/jamanetworkopen.2022.31973. PMC9486454 36121653

[ref36] Schwartz JB (2016) Potential effect of substituting estimated glomerular filtration rate for estimated creatinine clearance for dosing of direct oral anticoagulants. Journal of the American Geriatrics Society 64, 1996–2002. 10.1111/jgs.14288. 27549687

[ref37] Shakir A , Khan A , Agarwal S , Clifton S , Reese J , Munir MB , Nasir UB , Khan SU , Gopinathannair R , DeSimone CV , Deshmukh A , Jackman WM , Stavrakis S and Asad ZUA (2022) Dual therapy with oral anticoagulation and single antiplatelet agent versus monotherapy with oral anticoagulation alone in patients with atrial fibrillation and stable ischemic heart disease: a systematic review and meta-analysis. *Journal of Interventional Cardiac Electrophysiology* [Preprint]. 10.1007/s10840-022-01347-1. 36085242

[ref38] Shantsila E , Wolff A , Lip GY and Lane DA (2015) Optimising stroke prevention in patients with atrial fibrillation: application of the GRASP-AF audit tool in a UK general practice cohort. British Journal of General Practice 65, e16–e23. 10.3399/bjgp15X683113. PMC4276003 25548312

[ref39] Steffel J , Collins R , Antz M , Cornu P , Desteghe L , Haeusler KG , Oldgren J , Reinecke H , Roldan-Schilling V , Rowell N , Sinnaeve P , Vanassche T , Potpara T , Camm AJ , Heidbüchel H and External reviewers (2021) 2021 European heart rhythm association practical guide on the use of non-vitamin K antagonist oral anticoagulants in patients with atrial fibrillation. Europace 23, 1612–1676. 10.1093/europace/euab065. 33895845 PMC11636576

[ref40] Steinberg BA , Shrader P , Thomas L , Ansell J , Fonarow GC , Gersh BJ , Kowey PR , Mahaffey KW , Naccarelli G , Reiffel J , Singer DE , Peterson ED , Piccini JP and ORBIT-AF Investigators and Patients (2016) Off-label dosing of non-vitamin k antagonist oral anticoagulants and adverse outcomes. Journal of the American College of Cardiology 68, 2597–2604. 10.1016/j.jacc.2016.09.966. 27978942

[ref41] Steinberg BA , Shrader P , Pieper K , Thomas L , Allen LA , Ansell J , Chan PS , Ezekowitz MD , Fonarow GC , Freeman JV , Gersh BJ , Kowey PR , Mahaffey KW , Naccarelli GV , Reiffel JA , Singer DE , Peterson ED , Piccini JP and Outcomes Registry for Better Informed Treatment of Atrial Fibrillation (ORBIT‐AF) II Investigators (2018) Frequency and outcomes of reduced dose non–vitamin k antagonist anticoagulants: results from ORBIT-AF II (The outcomes registry for better informed treatment of atrial fibrillation II). Journal of the American Heart Association 7, e007633. 10.1161/JAHA.117.007633. 29453305 PMC5850192

[ref42] Thompson LE , Maddox TM , Lei L , Grunwald GK , Bradley SM , Peterson PN , Masoudi FA , Turchin A , Song Y , Doros G , Davis MB and Daugherty SL (2017) Sex differences in the use of oral anticoagulants for atrial fibrillation: a report from the National Cardiovascular Data Registry (NCDR ^®^) PINNACLE Registry. Journal of the American Heart Association 6, e005801. 10.1161/JAHA.117.005801. 28724655 PMC5586299

[ref43] Tzeis S , Savvari P , Skiadas I , Patsilinakos S , Stamatelopoulos K , Kourouklis S , Kyrikos S , Tsatiris K , Menegas D , Hahalis G , Giannakoulas G and PAVE-AF study group (2021) Right drug, wrong dosage: insights from the PAVE-AF antithrombotic study in older patients with atrial fibrillation. Journal of Thrombosis and Thrombolysis 51, 81–88. 10.1007/s11239-020-02167-8. 32519166 PMC7829237

[ref3] van Belle G (2008) Statistical rules of thumb, Second Edition. Wiley Series in Probability and Statistics. Hoboken, NJ, USA: John Wiley & Sons, Inc. 10.1002/9780470377963.

[ref33] van Rein N , Heide-Jørgensen U , Lijfering WM , Dekkers OM , Sørensen HT and Cannegieter SC (2019) Major bleeding rates in atrial fibrillation patients on single, dual, or triple antithrombotic therapy: results from a nationwide Danish cohort study. Circulation 139, 775–786. 10.1161/CIRCULATIONAHA.118.036248. 30586754

[ref44] Voskoboinik A , Kalman JM , De Silva A , Nicholls T , Costello B , Nanayakkara S , Prabhu S , Stub D , Azzopardi S , Vizi D , Wong G , Nalliah C , Sugumar H , Wong M , Kotschet E , Kaye D , Taylor AJ and Kistler PM (2020) Alcohol abstinence in drinkers with atrial fibrillation. New England Journal of Medicine 382, 20–28. 10.1056/NEJMoa1817591. 31893513

[ref45] Wijtvliet EPJP , Tieleman RG , van Gelder IC , Pluymaekers NAHA , Rienstra M , Folkeringa RJ , Bronzwaer P , Elvan A , Elders J , Tukkie R , Luermans JGLM , Van Asselt ADIT , Van Kuijk SMJ , Tijssen JG , Crijns HJGM and RACE 4 Investigators (2020) Nurse-led vs. usual-care for atrial fibrillation. European Heart Journal 41, 634–641. 10.1093/eurheartj/ehz666. 31544925

[ref46] Xian Y , O'Brien EC , Liang L , Xu H , Schwamm LH , Fonarow GC , Bhatt DL , Smith EE , Olson DM , Maisch L , Hannah D , Lindholm B , Lytle BL , Pencina MJ , Hernandez AF and Peterson ED (2017) Association of preceding antithrombotic treatment with acute ischemic stroke severity and in-hospital outcomes among patients with atrial fibrillation. JAMA 317, 1057. 10.1001/jama.2017.1371. 28291892

[ref47] Xie CX , Robson J , Williams C , Carvalho C , Rison S and Raisi-Estabragh Z (2022) Dual antithrombotic therapy and gastroprotection in atrial fibrillation: an observational primary care study. *BJGP Open*, BJGPO.2022.0048. 10.3399/BJGPO.2022.0048. PMC9904777 36028299

[ref48] Yao X , Shah ND , Sangaralingham LR , Gersh BJ and Noseworthy PA (2017) Non–vitamin K antagonist oral anticoagulant dosing in patients with atrial fibrillation and renal dysfunction. Journal of the American College of Cardiology 69, 2779–2790. 10.1016/j.jacc.2017.03.600. 28595692

[ref49] Yasuda S , Kaikita K , Akao M , Ako J , Matoba T , Nakamura M , Miyauchi K , Hagiwara N , Kimura K , Hirayama A , Matsui K , Ogawa H and AFIRE Investigators (2019) Antithrombotic therapy for atrial fibrillation with stable coronary disease. New England Journal of Medicine 381, 1103–1113. 10.1056/NEJMoa1904143. 31475793

